# Positive end-expiratory pressure can increase brain tissue oxygen pressure in hypoxemic severe traumatic brain injury patients

**DOI:** 10.1186/cc10189

**Published:** 2011-06-22

**Authors:** SN Nemer, R Santos, J Caldeira, P Reis, B Guimarães, T Loureiro, R Ramos, E Farias, D Prado, R Turon

**Affiliations:** 1 Hospital de Clínicas de Niterói, Niterói - RJ, Brazil

## Introduction

Brain tissue oxygen pressure (PtiO_2_) reflects brain oxygenation and is a useful tool in traumatic brain injury (TBI) patients. Increases in inspired oxygen fraction (FiO_2_) are related to improvement on PbrO_2_, but other approaches that aim to improve oxygenation, like increasing positive-end expiratory pressure (PEEP), were not deeply evaluated in humans.

## Objective

The aim of this study was to evaluate the effects of three different PEEP levels on PbrO_2 _of hypoxemic severe TBI patients.

## Methods

From February 2007 to February 2011, 36 severe TBI patients admitted to our intensive neurological unit were monitored with PtiO_2 _through the Licox device (Integra Neuroscience). Seventeen patients remained in the study according to the following inclusion criteria: ratio of arterial oxygen tension to fraction of inspired oxygen (PaO_2_/FiO_2 _ratio) <300; cerebral perfusion pressure (CPP) >60 mmHg; intracranial pressure (ICP) <20 mmHg; PtiO_2 _>20 mmHg; absence of any signal of brain deterioration. These patients were submitted to PEEP levels of 5, 10 and 15 cmH_2_O, each one for at least 20 minutes. During the three PEEP levels, PtiO_2_, pulse oxygen saturation (SpO_2_), ICP and CPP were monitored and statistically analyzed by ANOVA and Bonferroni methods. *P *< 0.05 was considered statistically significant.

## Results

**Figure 1:**
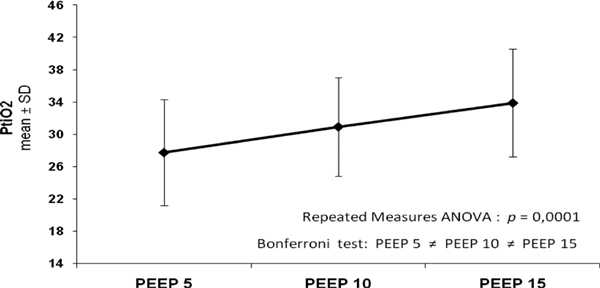
**Changes in PtiO_2 _according to the PEEP levels**.

**Figure 2:**
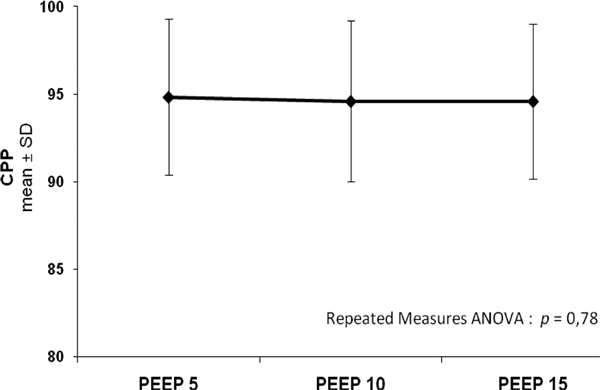
**Changes in CPP according to the PEEP levels**.

The increase of PEEP level from 5 to 15 cmH_2_O increased SpO_2 _from 95.5 ± 2.1 to 98.6 ± 1.2 (*P *= 0.0001) and PtiO_2 _from 27.8 ± 6.5 mmHg to 33.9 ± 6.7 mmHg, respectively (*P *= 0.0001). On the other hand, ICP and CPP did not present statistical significance according to the increase of PEEP levels (8.29 ± 4.44 mmHg to 8.65 ± 4.42 mmHg; *P *= 0.14 and 94.8 ± 8.2 to 94.6 ± 8.0 mmHg; *P *= 0.78, respectively). The main characteristics of the evaluated patients are described in Table 1. Changes in PtiO_2 _and CPP according to the PEEP levels are represented in Figures 1 and 2.

**Table 1:** Baseline characteristics of the evaluated patients

Baseline characteristic	Mean	SD
Age	28.6	8.4
APACHE II	19.2	3.2
Glasgow	6.1	0.9
FiO_2_	55.9	11.8
PaO_2_/FiO_2 _ratio	154	46.6
PbrO_2_	27.7	6.5
ICP	8.3	4.4
CPP	94.8	8.2
SpO_2_	95.5	2.1

APACHE II, Acute Physiology and Chronic Health Evaluation.

## Conclusion

In hypoxemic severe TBI patients, increasing PEEP levels from 5 to 10 and 15 cmH_2_O increased PtiO_2_, without increasing ICP and/or decreasing CPP. Increasing PEEP levels can be an alternative ventilatory approach to improve brain oxygenation besides FiO_2_.

